# Serological Investigation of Vincent’s Angina

**Published:** 1919

**Authors:** 


***Serological Investigation of Vincent's Angina.*** By F. E. Taylor and W. H. MacKinstry. *Journal of Laryngology, Rhinology and Otology*, October, 1918.

The question of the syphilitic origin of Vincent's angina is discussed by the authors, who quote a series of contradictory opinions expressed on this subject by various bacteriologists, and give the results of their own observations made on several hundreds of cases of sore throat.

In cases of Vincent's angina, Wassermann's test was found to be positive by some observers like St. Clair Thomson, Gerber, Moch and Soberheim; others, such as Bouty, Campbell, Dyas and Bowmann obtained negative results. Erlich recommends intravenous Salvarsan injections, while Weinstein and Saverio maintain that no definite opinion can be formed at present from the data at hand.

Over 300 cases of fuso-spirillary infection, including approximately 150 typical cases of Vincent's angina, were examined bacteriologically at Queen Alexandra's Military Hospital — Millbank. From this number, 55 cases were taken at random and had their blood tested by Wassermann's reaction. In all of these cases but two the reaction was negative, and in the two positive cases the patients suffered from latent syphilis.

The conclusion arrived at by the authors may be summarized as follows: The prevailing belief in the occurrence of a positive Wassermann reaction in Vincent's angina has no foundation; the two conditions can be differentiated with certainty by the application of bacteriological and serological methods. When the complement-fixation test of Wassermann is positive, there is double infection, either a coincident syphilitic and Vincent's infection, or Vincent's angina in a subject with latent syphilis.

